# A Highly Sensitive Nonenzymatic Glucose Biosensor Based on the Regulatory Effect of Glucose on Electrochemical Behaviors of Colloidal Silver Nanoparticles on MoS_2_^†^

**DOI:** 10.3390/s17081807

**Published:** 2017-08-05

**Authors:** Kash Anderson, Benjamin Poulter, John Dudgeon, Shu-En Li, Xiang Ma

**Affiliations:** 1 Department of Chemistry, Idaho State University, Pocatello, ID 83201, USA; andekas2@isu.edu (K.A.); poulbenj@isu.edu (B.P.); lishue@isu.edu (S.-E.L.); 2 Department of Anthropology, Idaho State University, Pocatello, ID 83201, USA; dudgeon@isu.edu; 3 Present address: Department of Chemistry, Grand View University, Des Moines, IA 50316, USA

**Keywords:** glucose biosensor, nonenzymatic, colloidal silver nanoparticle, molybdenum disulfide

## Abstract

A novel and highly sensitive nonenzymatic glucose biosensor was developed by nucleating colloidal silver nanoparticles (AgNPs) on MoS_2_. The facile fabrication method, high reproducibility (97.5%) and stability indicates a promising capability for large-scale manufacturing. Additionally, the excellent sensitivity (9044.6 μA·mM^−1^·cm^−2^), low detection limit (0.03 μM), appropriate linear range of 0.1–1000 μM, and high selectivity suggests that this biosensor has a great potential to be applied for noninvasive glucose detection in human body fluids, such as sweat and saliva.

## 1. Introduction

As of 2015, 415 million people suffer from diabetes worldwide, and 318 million people are at high risk of developing the disease in the future [1]. Diabetes is a chronic medical condition in which levels of glucose in the blood are raised significantly from the normal range. The high levels of glucose in the blood will eventually cause damage to many tissues in the body, including heart, eyes, kidneys and nerves, leading to painful and life-threatening health complications. According to the International Diabetes Federation, every six seconds a person dies from the health complications associated with diabetes. However, these complications can be prevented or controlled by monitoring blood glucose levels. Accordingly, millions of diabetics test their blood glucose levels daily, which makes glucose the most commonly tested analyte [2].

Since the first invention of glucose enzyme electrodes by Clark and Lyons in 1962 [3], a tremendous amount of research has been dedicated in developing enzyme-based biosensors [4,5,6,7], which rely on the catalytic activity of glucose oxidase (GOx) towards glucose in the blood [8,9,10,11,12,13,14,15,16,17,18,19]. However, this strategy has two fundamental drawbacks: first, the painful blood drawing process may prevent patients from their daily (or more frequently) blood sample testing; and second, the GOx enzyme is relatively fragile and expensive. Recent studies have showed a strong correlation between the glucose levels in human blood and those in other body fluids, such as sweat [20] and saliva [21]. For example, sweat glucose that was properly harvested to prevent contamination from other sources on the skin accurately reflected the blood glucose levels of patients [20]. The concentrations of glucose in these biofluids are in the μM range [20,22]. Thus, the development of nonintrusive, inexpensive and nonenzymatic glucose biosensors that are sensitive enough to effectively detect glucose in alternative body fluids of sweat and saliva is in great need.

MoS_2_ (molybdenum disulfide), a two-dimensional material with large surface area, rich surface chemistry, excellent biocompatibility, and a weakly bonded and layered structure, has attracted scientists’ attention in a variety of fields in recent years [23,24,25,26,27,28,29,30,31,32,33]. While the unique atomic structure of MoS_2_ allows easy intercalation of metal atoms or ions [34,35], MoS_2_-based electrochemical devices suffer from unsatisfactory performance due to its poor intrinsic conductivity. As such, conductive additives have been employed to improve the electrochemical performance of MoS_2_ [36,37,38]. In this context, introducing noble metal nanoparticles, such as silver [39], as a conductive additive shall address this inadequacy and achieve an improved electrochemical performance. 

Here, colloidal AgNPs were synthesized in the presence of MoS_2_, which was used to fabricate highly sensitive, nonenzymatic biosensors for the detection of relatively low glucose levels in human sweat and saliva. To our best knowledge, this is the first report of colloidal AgNPs/MoS_2_-based nonenzymatic glucose biosensor.

## 2. Materials and Methods 

### 2.1. Reagents and Materials

Silver nitrite (>99% AgNO_3_), sodium borohydride (99% NaBH_4_), glucose, glycine, urea, l-phenylalanine, l-lactic acid and l-tyrosine were purchased from Sigma-Aldrich (St. Louis, MO, USA). MoS_2_ powder (10–30 μm) was obtained from Rose Mill Co. (West Hartford, CT, USA). All other reagents and chemicals were of analytical grade and were used as received without further purification.

### 2.2. Preparation of AgNPs/MoS_2_ Modified Electrodes

The MoS_2_ powder was added to deionized water at an initial concentration of 50 mg·mL^−1^, and then subjected to sonication at 500 W for 60 min. The obtained slurry was set overnight. Then the supernatant was collected and mixed with AgNO_3_. The mixture, containing 1.0 mM AgNO_3_, was added dropwise to 2.0 mM NaBH_4_ that had been cooled in an ice bath, while stirred vigorously. When the solution turned light yellow, any further addition of the mixture and stirring was stopped immediately. The clear yellow solution was stable at room temperature when stored in a sealed vial in the dark for several months. The yellow solution of AgNPs/MoS_2_ was used for electrode modification and subjected to structural characterizations later.

The AgNPs/MoS_2_ modified electrode was fabricated by coating various amounts of AgNPs/MoS_2_ solution onto the surface of polished glassy carbon (GC) electrodes. 20 μL of AgNPs/MoS_2_ gave the highest electrochemical response. After an overnight drying process, the electrode was carefully rinsed with water, dried again at room temperature, and then used for electrochemical measurements.

### 2.3. Electrochemical Measurements

Electrochemical measurements were performed on a PGSTAT204 electrochemical workstation (Metrohm, Houston, TX, USA) in a three-electrode electrochemical cell at room temperature of 25 °C. The AgNPs or AgNPs/MoS_2_-modified GC electrode was used as the working electrode, Ag/AgCl as the reference electrode, and Pt wire as the counter electrode. CVs (cyclic voltammetry) were obtained with a potential window of −0.50 to 0.50 V (vs. Ag/AgCl) in 0.1 M NaOH solution. SWV (square wave voltammetry) measurements were carried out with a potential window of 0.10–0.60 V (vs. Ag/AgCl) in 0.1 M NaOH solution at a frequency of 10 Hz. A magnetic stirring was applied to the solution during SWV measurements to achieve convective mass transfer.

### 2.4. Materials Characterization

UV-Vis absorption spectroscopic measurements were performed using Evolution 300 UV-Vis spectrophotometer (Thermo Fisher Scientific, Waltham, MA, USA). The morphology of the AgNPs/MoS_2_ modified electrode was determined using scanning electron microscopy (SEM) (LEO 1430 VP, Carl Zeiss, Oberkochen, Germany), energy-dispersive X-ray (EDS) analysis, and atomic force microscopy (AFM) (Nexus One, NT-MDT, Tempe, AZ, USA). AFM measurements were performed in AC mode in 0.1 M NaOH solution with and without 1 mM glucose at room temperature on freshly cleaved mica.

### 2.5. Real Sample Collection and Preparation

One male participant was recruited for this study. Written consent was sought prior to commencement of the study and ethical approval was provided by the Human Subjects Committee, Idaho State University, US (Reference IRB-FY2017-304).

Saliva samples (~1 mL) were collected using a passive drool method without stimulation. Samples were collected into plastic vials and stored at −20 °C until analysis. After thawing and centrifugation, the samples were diluted 1:1 volume ratio with 0.2 M NaOH solutions so that the final concentration of NaOH was 0.1 M. 0.1 M NaOH was chosen as a standard testing solution, because it removes concerns about the impact of pH and ionic strength in different samples on the performance of the biosensor. However, alkaline solution may denature the biomolecules in samples, which may interfere with reliable detections of glucose. If large amount of denatured biomolecules, such as proteins, are observed, further purification steps (centrifugation, liquid chromatography, etc.) may be necessary.

## 3. Results

### 3.1. Materials Characterization of the Biosensor

**Figure 1:**
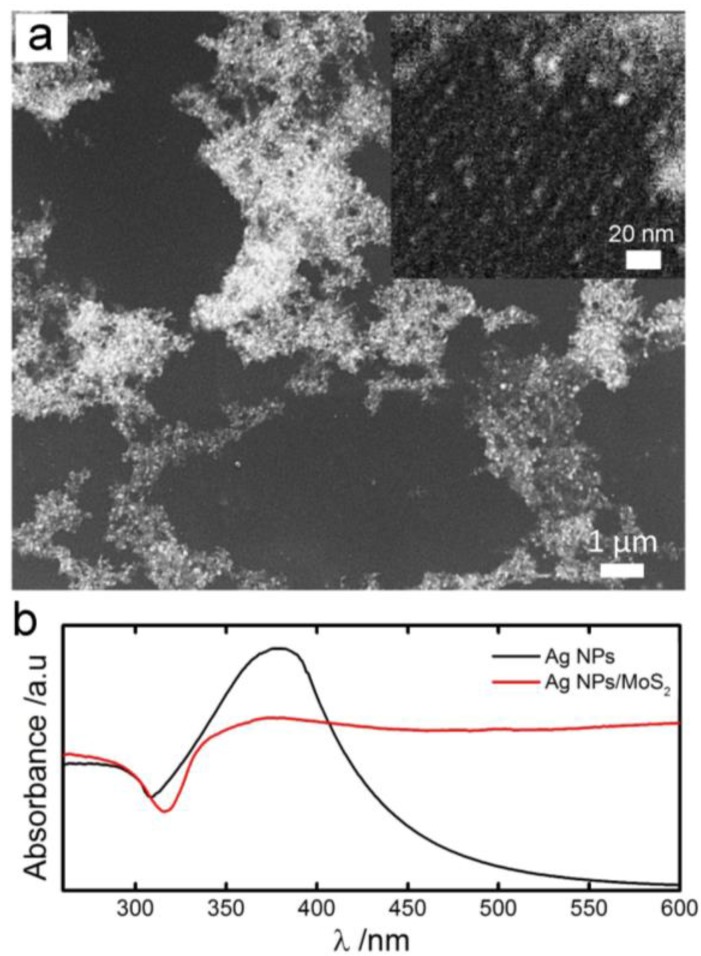
Materials Characterization. (**a**) Scanning electron microscopy (SEM) images of colloidal AgNPs nucleated on MoS_2_; (**b**) UV-Vis spectrum of AgNPs and AgNPs/MoS_2_.

SEM images of AgNPs/MoS_2_ showed that AgNPs exhibited a three-dimensional porous network structure (Figure 1a). Clusters of AgNPs, with a diameter of 1–7 μm, distributed unevenly on the surface. The size of the clusters was comparable to that of MoS_2_ flakes (~10 μm). The average diameter of AgNPs within the clusters was estimated to be ~5 nm, which was supported by UV-Vis spectrum (Figure 1b): the wavelength of the plasmon absorption maximum was near 390 nm, indicating a particle size of 5 nm [40]. The EDS spectrum of the AgNPs/MoS_2_ film confirmed the co-existence of Ag and Mo (molecular weight ratio of Ag/Mo is 20:1), suggesting that AgNPs were nucleated on MoS_2_ layers. In solution AFM images also showed that AgNPs were firmly attached on MoS_2_, or otherwise, they would not be observed. (Figure S1).

### 3.2. Electrochemical Behavior

**Figure 2:**
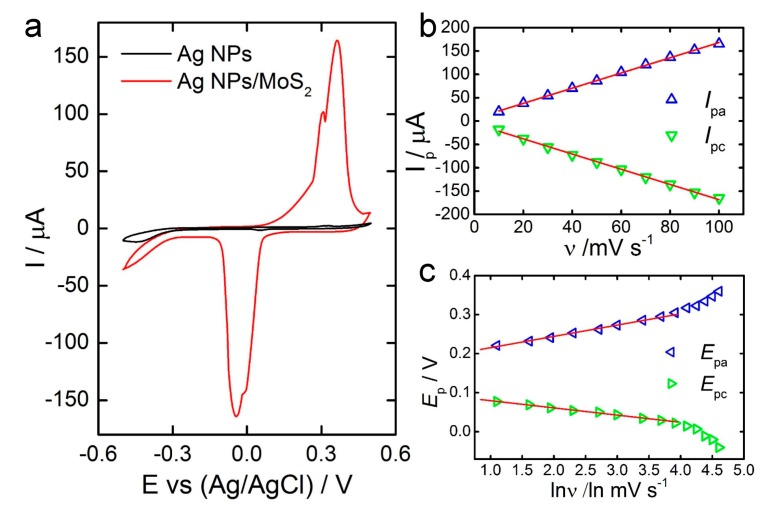
Electrochemical Characterization. (**a**) CV (cyclic voltammetry) curves of AgNPs and AgNPs/MoS_2_ electrodes in 0.1 M NaOH at a scan rate of 0.1 V·s^−1^; (**b**) Peak currents versus scan rate (*ν*); (**c**) Peak potential versus ln *ν*.

Cyclic voltammograms (CVs) of the AgNPs and AgNPs/MoS_2_ electrodes showed an enhanced electrochemical reactivity of AgNPs/MoS_2_ compared with AgNPs only (Figure 2a). Two anodic peaks at 0.31 and 0.36 V (vs. Ag/AgCl) may represent the oxidation of Ag to Ag^+^ and Ag^+^ to Ag^2+^, while two cathodic peaks at −0.01 and −0.04 V may be associated with the reversible conversion of Ag^2+^ back to Ag. The electrochemical redox phenomena of Ag/Ag^+^/Ag^2+^ which occur at the working electrode surface in a basic solution can be represented as follows:

Ag + OH^−^ ↔ AgOH + e^−^ (1)

AgOH + OH^−^ ↔ Ag(OH)_2_ + e^−^ (2)

Ag^2+^ normally requires a higher potential than 0.36 V to be generated. Here, we attribute the electrochemical signal at 0.36 V to the oxidation of Ag^+^ to Ag^2+^, because when the size of AgNPs comes to the rage of ~10 nm, the activation energy required for the oxidation of Ag^+^ to Ag^2+^ may be significantly reduced, potentially due to a decrease in interfacial energy and an increase in the portion of surface/interface atoms. However, other mechanistic representations may also be considered.

The potential scan rate (*υ*) was observed to be linearly related to the currents of both oxidation and reduction peaks (Figure 2b), a characteristic of diffusionless, thin-layer electrochemical behavior. The linear regression equations, y = 5.3636 + 1.6275x and y = −6.0773 − 1.6197x, and the regression coefficients, 0.9995 and 0.9987, were obtained for oxidation and reduction peaks, respectively. In addition, the *E_pa_* and *E_pc_* are linearly dependent on the logarithm of the scan rate (Figure 2c). Laviron’s model [41] gives:

(3) $$E_{pa}=E^{\theta }+\;\frac{RT}{\left(1-\alpha \right)nF}ln\frac{\left(1-\alpha \right)Fn\upsilon }{RTK_{s}}$$

(4) $$E_{pc}=\;E^{\theta }-\;\frac{RT}{\alpha nF}ln\frac{\alpha Fn\upsilon }{RTK_{s}}$$

(5) $$logk_{s}=\alpha \log\left(1-\alpha \right)+\left(1-\alpha \right)log\alpha -log\frac{RT}{nF\upsilon }\;-\;\frac{\alpha \left(1-\alpha \right)nF∆E_{p}}{2.3RT}$$

where *n* is the number of electrons transferred in the rate determining step, ∆*E_p_* is the peak potential separation, *υ* is the scan rate, and *R*, *T*, and *F* are constants (*R* = 8.314 J·mol^−1^·K^−1^, *T* = 298 K, *F* = 96,485 C·mol^−1^). The electron-transfer coefficient (*α*) and apparent charge-transfer constant (*k_s_*) were thereby calculated to be 0.68 and 17.55 s^−1^, respectively, indicating that electron transfer was effectively promoted on the AgNPs/MoS_2_ electrode. 

### 3.3. Glucose Detection

**Figure 3:**
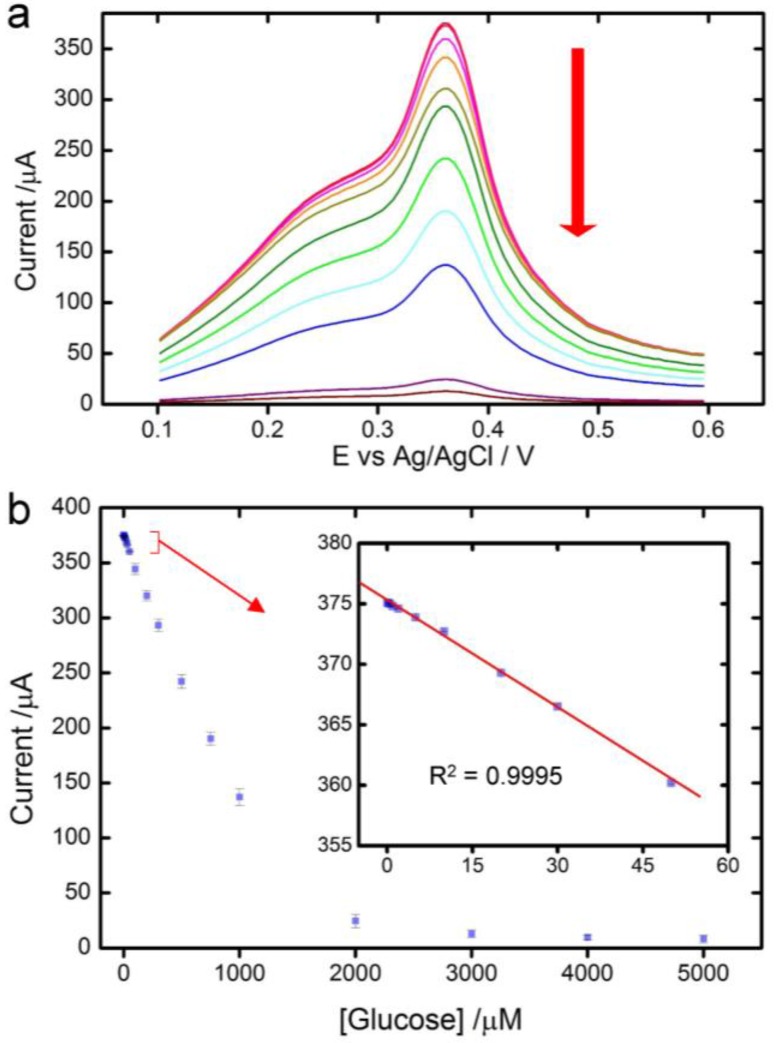
Glucose Detection. (**a**) Square wave voltammetry (SWV) curves of the AgNPs/MoS_2_ electrode in 0.1 M NaOH containing 0.1, 10, 50, 100, 200, 300, 500, 750, 1000 and 2000 μM glucose, at a scan rate of 0.1 V s^−1^; (**b**) The calibration curves of the biosensor.

Square wave voltammetry (SWV) was employed for the detection of glucose due to its high sensitivity and speed. After the electrode was stabilized in 0.1 M NaOH by running 10 times of SWV, solutions of glucose with different concentrations were added successively to the system. The current of the anodic peak decreased with increase of glucose concentrations (Figure 3a), suggesting an inhibitory effect of glucose on the electrochemical reactivity of the biosensor. This effect was reversible, as the biosensor regained its full electrochemical reactivity when the electrode was rinsed and inserted back into 0.1 M NaOH without glucose even after several repeated processes (Figure S2). We also observed that when the inhibitory effect was saturated (at a glucose concentration >4 mM), the current of the anodic peak reached 8.0 μA, similar to the one of the electrode modified with AgNPs only (Figure 2a). In-solution AFM images showed that the thickness of MoS_2_ layers increased when glucose was added (Figure S1). These results imply that the regulation of the electrochemical reactivity by glucose takes effect by separating MoS_2_ layers, which prevents them from facilitating electron transfers between AgNPs and the electrode. Further studies are needed to determine detailed mechanisms of glucose regulation.

The current-concentration curve (Figure 3b) showed a linear relationship in the range of 100 nM to 1 mM of glucose concentration, with a sensitivity of 9044.6 μA·mM^−1^·cm^−2^ (I (μA) = −0.284 × C (mM) + 375.14 (*R*^2^ = 0.9995)). The limit of detection (LoD) was estimated to be 0.03 μM (S/N = 3). This LoD is two orders lower than the glucose concentration in sweat and saliva in healthy patient, 8.3–120 μM [42,43]. Therefore, the AgNPs/MoS_2_ biosensor can be readily applied for noninvasive glucose detection in sweat and saliva. Both the sensitivity and LoD of the AgNPs/MoS_2_ biosensor are superior to those of previously reported enzymatic and nonenzymatic biosensors (Table 1). We attribute the enhanced performance of the biosensor to high conductivity of Ag, large surface-to-volume ratio of the AgNPs nanostructure, large surface area of MoS_2_ and the precise regulation of glucose on electrochemical behaviors of AgNPs on MoS_2_.

### 3.4. Selectivity, Stability and Reproducibility of the Biosensor

**Figure 4:**
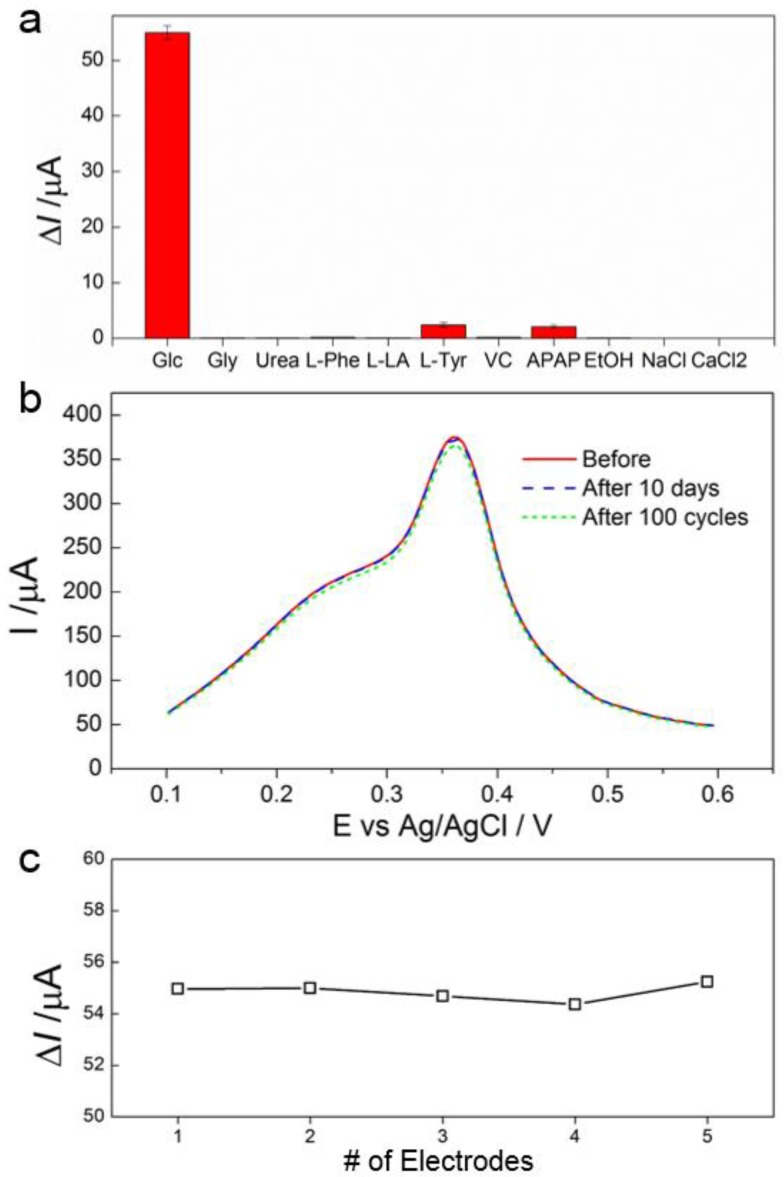
Selectivity, Stability and Reproducibility. (**a**) Changes of the peak current of the biosensor in the presence of 0.2 mM glucose, 0.02 mM Gly, urea, L-Phe, L-LA, L-Tyr, VC, APAP, EtOH and 0.2 mM NaCl and CaCl_2_; (**b**) SWV curves of the biosensor collected immediately after fabrication, after performing 100 CV cycles, and after being stored in the 4 °C refrigerator for 10 days, in 0.1 M NaOH; (**c**) Changes of the peak current recorded using five different biosensors in 0.1 M NaOH containing 0.2 mM glucose.

Selectivity of the AgNPs/MoS_2_ biosensor was evaluated by adding interfering substances commonly found in sweat and saliva, including 0.02 mM glycine (Gly), urea, l-phenylalanine (L-Phe), l-lactic acid (L-LA), l-tyrosine (L-Tyr), ascorbic acid (VC), paracetamol (APAP), alcohol (EtOH) and 0.2 mM NaCl and CaCl_2_ into the system. The biosensor showed no response towards Gly, urea, L-Phe, L-LA, NaCl and CaCl_2_, and only slight response (<5%) to L-Tyr and APAP (Figure 4a), indicating high selectivity of the biosensor towards glucose detection.

The stability and reproducibility of the biosensor were also evaluated. The stability was examined by recording 100 consecutive CV curves in 0.1 M NaOH, and after the electrode was stored in the 4 °C refrigerator for 10 days. No obvious change in peak current was observed (Figure 4b), indicating a good stability of the biosensor. The reproducibility was examined by testing five different AgNPs/MoS_2_ fabricated independently for their current response to glucose. From results shown in Figure 4c, a reproducibility rate of >97.5% is calculated, indicating that the biosensor is highly reproducible. 

### 3.5. Glucose Detection in Real Samples

The practical application of the biosensor was evaluated by adding glucose to human sweat or saliva samples. The recovery tests were performed and the results were listed in Table 2. The recoveries of the glucose concentration obtained by standard additions of glucose to real human samples ranged from 97% to 99.5%. These results suggest that the AgNPs/MoS_2_ biosensor can be used practically to detect glucose in real biological samples.

## 4. Discussion and Conclusions

A highly sensitive nonenzymatic glucose biosensor was developed using inexpensive fabrication method and biocompatible materials. The detection of glucose was achieved by a mechanism where glucose precisely regulates the electrochemical reactivity of AgNPs through potentially physical separation of AgNPs and MoS_2_. The porous nanostructures of AgNPs and large surface areas of MoS_2_ enhanced the interactive sites between AgNPs and electrode/glucose, contributing to accelerated electron transfer of AgNPs and the high sensitivity of the biosensor. The biosensor exhibited excellent sensitivity, stability and reproducibility, low LoD, and high selectivity, suggesting a novel candidate for noninvasive glucose monitoring for patients with diabetes. Further, the mechanism of action and facile fabrication method offer a novel approach for the development of other nonenzymatic biosensors and large-scale manufacturing. Future studies will be needed to employ the biosensor for in vivo and real time glucose detections.

## Figures and Tables

**Table 1:** Comparison of the performance of several electrochemical glucose biosensors.

Sensor	LoD (μM)	Sensitivity (μA·mM^−1^·cm^−2^)	Linear Range (μM)	Reference
GC/Colloidal AgNPs/MoS_2_	0.03	9044.6	0.1–1000	This work
GC/Ag-CNx	0.6	97	1–100	[44]
GC/Cu-Ag_2_O NWs	10	298.2	200–3200	[45]
CuNCs-DLEG	0.25	4532	25–4500	[46]
CuO NPs/Ag/Si	0.5	2762.5	50–18,450	[47]
Pt/AgTNPs/CHIT/GOx	1	67.17	3–3000	[48]
rGO/PAMAM/Ag/GOx	4.5	75.72	32–1890	[49]
AgNWs/CS/GOx	2.1	16.72	1000–15,000	[50]

**Table 2:** Detection of glucose in human samples.

Samples	Glucose Concentration of Samples (μM)	Glucose Added (μM)	Glucose Found (μM)	Recovery (%)	RSD (%)
Sweat	80.71	7	86.54	98.6	1.1
		100	179.82	99.5	1.1
Saliva	7.42	3	10.11	97.0	1.7
		20	27.02	98.5	1.3

## Supplementary Materials

The following are available online at www.mdpi.com/1424-8220/17/8/1807/s1, Figure S1: Reversibility of the Regulatory Effect. The ratio of the peak current of the AgNPs/MoS_2_ electrode after repeatedly performing glucose detection in the solution containing glucose and then being brought back to 0.1 M NaOH, to the initial peak current. Figure S2: Reversibility of the Regulatory Effect. The ratio of the peak current of the Ag NPs/MoS_2_ electrode after repeatedly performing glucose detection in the solution containing glucose and then being brought back to 0.1 M NaOH, to the initial peak current.
