# Supplementary material for: A Highly Sensitive Nonenzymatic Glucose Biosensor Based on the Regulatory Effect of Glucose on Electrochemical Behaviors of Colloidal Silver Nanoparticles on MoS2 †
Source: Sensors (Basel). 2017 Aug 5;17(8):1807. doi: 10.3390/s17081807 (PMC5579834; doi:10.3390/s17081807)
Supplement: Supplementary file 1 [file sensors-17-01807-s001.pdf]

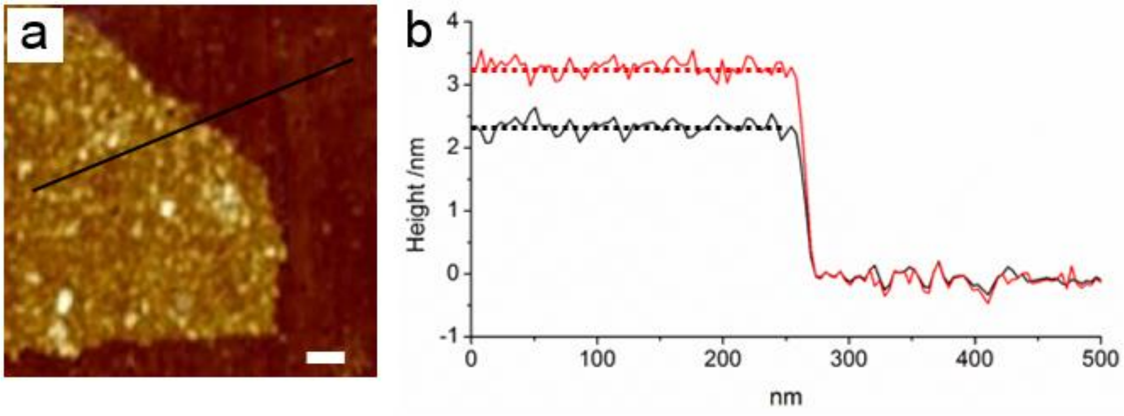

Figure S1. Atomic Force Microscopy (AFM) Characterization. (**a**) In-solution AFM image of AgNPs/MoS_2. Z scale: 4.5 nm. Scale bar: 50 nm; (**b**) Height information of cross section in a shows that the height of MoS_2 increased by ~1 nm when 1 mM glucose was added in the solution (black line: no glucose, red line: 1 mM glucose).

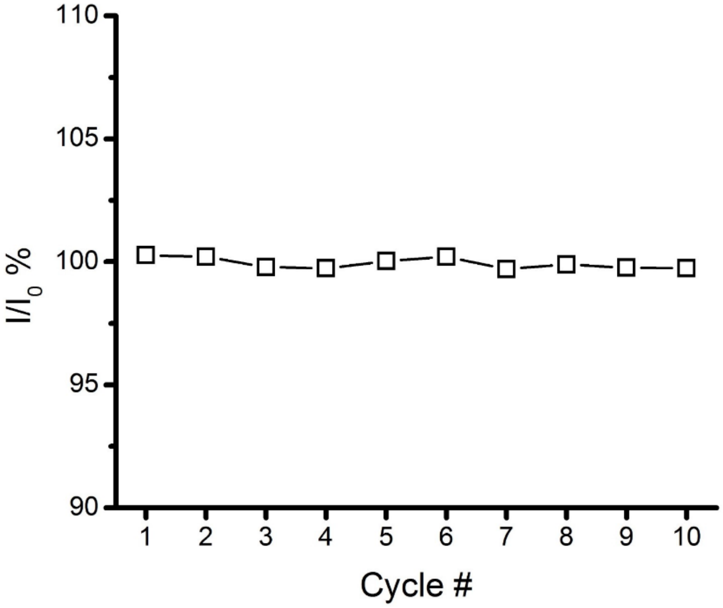

Figure S2. Reversibility of the Regulatory Effect. The ratio of the peak current of the AgNPs/MoS_2 electrode after repeatedly performing glucose detection in the solution containing glucose and then being brought back to 0.1 M NaOH, to the initial peak current.
